# Updated Operational Guidance for Implementing CDC’s Recommendations on Testing for Hepatitis C Virus Infection

**DOI:** 10.15585/mmwr.mm7228a2

**Published:** 2023-07-14

**Authors:** Emily J. Cartwright, Priti Patel, Saleem Kamili, Carolyn Wester

**Affiliations:** ^1^Division of Viral Hepatitis, National Center for HIV, Viral Hepatitis, STD, and TB Prevention, CDC; ^2^Emory University, Atlanta, Georgia; ^3^Atlanta VA Health Care System, Decatur, Georgia.

**Summary**

**What is known about this topic?**

Current hepatitis C virus (HCV) testing guidance recommends a two-step testing sequence for diagnosis of HCV infection. When an HCV antibody test is reactive and no HCV RNA test is performed, testing is considered incomplete. Historically, approximately one third of patients have incomplete testing.

**What is added by this report?**

New guidance for completion of HCV testing supports operational strategies that collect samples at a single visit, and automatic HCV RNA testing on all HCV antibody reactive samples. Use of strategies that require multiple visits to collect samples should be discontinued.

**What are the implications for public health practice?**

Automatic HCV RNA testing on all HCV antibody reactive samples will increase the percentage of patients with current HCV infection who are linked to care and receive curative antiviral therapy.

## Abstract

Current hepatitis C virus (HCV) testing guidance recommends a two-step testing sequence for diagnosis of HCV infection. Performing an HCV RNA test whenever an HCV antibody test is reactive (complete testing) is critical to achieve national HCV elimination goals. When an HCV antibody test is reactive and no HCV RNA test is performed, testing is considered incomplete. Historically, approximately one third of patients have incomplete testing. This update clarifies that all sites performing HCV screening should ensure single-visit sample collection. This approach allows for automatic HCV RNA testing when an HCV antibody test is reactive to avoid incomplete testing. Use of strategies that require multiple visits to collect HCV testing samples should be discontinued. Automatic HCV RNA testing on all HCV antibody reactive samples will increase the percentage of patients with current HCV infection who are linked to care and receive curative antiviral therapy. 

## Introduction

Examination of the hepatitis C care cascade in the United States reveals a substantial gap between the number of persons who have a reactive hepatitis C virus (HCV) antibody test and those who undergo nucleic acid testing (NAT) for detection of HCV RNA (*1*). Performing an HCV RNA test whenever an HCV antibody test is reactive (complete testing) is critical to increase the percentage of patients diagnosed with current HCV infection who are linked to care and receive curative antiviral therapy. To address the challenge of incomplete hepatitis C testing, many laboratories have implemented automatic HCV RNA testing whenever an HCV antibody test result is reactive (*2*–*4*). “Automatic” testing refers to laboratory testing that occurs without additional action on the part of the patient or the health care provider. 

### Testing for Hepatitis C Virus

Persons with a reactive HCV antibody test result and detectable HCV RNA are determined to have current HCV infection and should be linked to care. Persons who received a reactive HCV antibody test result and undetectable HCV RNA likely have a resolved HCV infection, although falsely reactive HCV antibody tests can occur (*5*). The 2013 CDC testing guidance* describes four possible operational strategies to diagnose current HCV infection:

1. Blood from a subsequent venipuncture is submitted for HCV RNA testing if the blood sample collected is reactive for HCV antibody during initial testing;
2. From a single venipuncture, two specimens are collected in separate tubes, one tube for initial HCV antibody testing, and a second tube for HCV RNA testing if the HCV antibody test is reactive;
3. The same sample of venipuncture blood used for initial HCV antibody testing, if reactive, is reflexed for HCV RNA testing without another blood draw; and
4. A separate blood sample is submitted for HCV RNA testing if the initial testing of HCV antibody has used finger-stick blood.

Operational strategies 2–4 allow for single-visit sample collection, which ensures that HCV RNA testing is performed automatically without requiring a separate health care visit. Operational strategy 1, however, requires two visits to a health care facility, and therefore leads to missed opportunities for HCV diagnosis and linkage to curative HCV treatment.

## Methods

In October 2021, the Association of Public Health Laboratories convened a meeting with experts from public health laboratories, academic medical centers, commercial laboratories, public health agencies, and community-based organizations to discuss obstacles to HCV testing in the United States.^†^ After the meeting, CDC reviewed the published literature to determine the magnitude of incomplete hepatitis C testing using the two-step testing sequence.

### Review of the Evidence

The following studies conducted in a variety of settings found that use of operational strategy 1 resulted in a sizable proportion of persons having incomplete HCV testing. In addition, studies have found that complete testing rates improve when operational strategies 2–4 are implemented. For example, data from the Chronic Hepatitis Cohort Study found that only 62% of patients had complete HCV testing (*6*). Similarly, only 66% of HCV antibody reactive patients who reported to the New York City Department of Health and Mental Hygiene surveillance system had complete HCV testing; this prompted a requirement in 2015 that all laboratories perform automatic HCV RNA testing (operational strategies 2–4) (*7*). Among Veterans Health Administration (VA) facilities that required a separate visit for subsequent HCV RNA testing (operational strategy 1), only 64% of patients completed the HCV testing sequence, whereas 98% of veterans completed testing in facilities that used operational strategies 2–4 (*8*). Since 2018, VA directive 1300.01 has required that all specimens that are reactive for HCV antibody undergo automatic testing for HCV RNA. Similarly, the Cherokee Nation Health Services found that 68% of persons had complete HCV testing when using operational strategy 1, but after implementing automatic HCV RNA testing, the proportion with complete testing increased to 85% (*2*,*9*). The Mid-Atlantic Permanente Medical Group developed a multifaceted hepatitis C care pathway that included automatic HCV RNA testing and found that the diagnosis of current HCV infection was statistically significantly higher when using the hepatitis C care pathway compared with the historical approach that used operational strategy 1 (*3*). Operational strategy 1 has also been found to not be cost-effective (*10*).

### Updated Operational Guidance

This update clarifies that operational strategy 1 should be discontinued; operational strategies 2, 3, or 4 should be used to diagnose current HCV infection. In settings where HCV antibody testing is performed using finger-stick blood (operational strategy 4), a separate sample should be collected at the same visit to ensure that HCV RNA testing is completed when the HCV antibody result is reactive. If an HCV antibody is reactive and no HCV RNA test is performed, testing is considered incomplete; an HCV RNA test should be performed for all HCV antibody reactive samples to establish the diagnosis of current HCV infection. Sites performing HCV screening should ensure single-visit sample collection (operational strategies 2–4) are used to avoid incomplete HCV testing.

## Discussion

Complete and accurate testing is the first step in identifying persons with current HCV infection to ensure linkage to care and initiation of curative antiviral therapy. Operational strategy 1 should no longer be used because it can lead to incomplete HCV testing and gaps in the HCV care cascade. Health care facilities and laboratories should update practices to ensure operational strategy 1 is no longer used. Using a single visit to conduct both steps of the HCV testing sequence will increase complete diagnosis of current HCV infection, which will increase the percentage of patients with current HCV infection who are linked to care and receive curative antiviral therapy.

## Footnotes

*
https://www.cdc.gov/mmwr/pdf/wk/mm62e0507a2.pdf

†
https://www.aphl.org/programs/infectious_disease/Documents/2022_05_APHL_HCV_Elimination_Meeting_Report.pdf
